# Sir Joseph Lister’s Operations

**Published:** 1887-01

**Authors:** 


### Sir Joseph Lister's Operations.

The foreign correspondent of the *American Practitioner and News*, who has watched Sir Joseph Lister operate, says that he is excessively slow, and is by no means careful in the details of antisepsis. He has given up the spray entirely, and is now using for dressings gauze and cotton impregnated with a new antiseptic. The nature of this is at present a secret. The bold Kentucky correspondent confesses that he "had the cheek to ask Sir Joseph for information on the subject, when he very politely replied that he was sorry to have to decline giving it to me; that it was yet a secret, and he wished to keep it as such until he was fully satisfied as to its efficacy, when he would make it known to the profession. He said that it had been on trial in his wards for many months, and that so far he was greatly pleased with it."

